# A structural analysis of *in vitro *catalytic activities of hammerhead ribozymes

**DOI:** 10.1186/1471-2105-8-469

**Published:** 2007-11-30

**Authors:** Yu Shao, Susan Wu, Chi Yu Chan, Jessie R Klapper, Erasmus Schneider, Ye Ding

**Affiliations:** 1 Wadsworth Center, New York State Department of Health, 150 New Scotland Avenue, Albany, NY 12208, USA

## Abstract

**Background:**

Ribozymes are small catalytic RNAs that possess the dual functions of sequence-specific RNA recognition and site-specific cleavage. *Trans*-cleaving ribozymes can inhibit translation of genes at the messenger RNA (mRNA) level in both eukaryotic and prokaryotic systems and are thus useful tools for studies of gene function. However, identification of target sites for efficient cleavage poses a challenge. Here, we have considered a number of structural and thermodynamic parameters that can affect the efficiency of target cleavage, in an attempt to identify rules for the selection of functional ribozymes.

**Results:**

We employed the Sfold program for RNA secondary structure prediction, to account for the likely population of target structures that co-exist in dynamic equilibrium for a specific mRNA molecule. We designed and prepared 15 hammerhead ribozymes to target GUC cleavage sites in the mRNA of the breast cancer resistance protein (BCRP). These ribozymes were tested, and their catalytic activities were measured *in vitro*. We found that target disruption energy owing to the alteration of the local target structure necessary for ribozyme binding, and the total energy change of the ribozyme-target hybridization, are two significant parameters for prediction of ribozyme activity. Importantly, target disruption energy is the major contributor to the predictability of ribozyme activity by the total energy change. Furthermore, for a target-site specific ribozyme, incorrect folding of the catalytic core, or interactions involving the two binding arms and the end sequences of the catalytic core, can have detrimental effects on ribozyme activity.

**Conclusion:**

The findings from this study suggest rules for structure-based rational design of *trans*-cleaving hammerhead ribozymes in gene knockdown studies. Tools implementing these rules are available from the Sribo module and the Srna module of the Sfold program available through Web server at .

## Background

**Figure 1:**
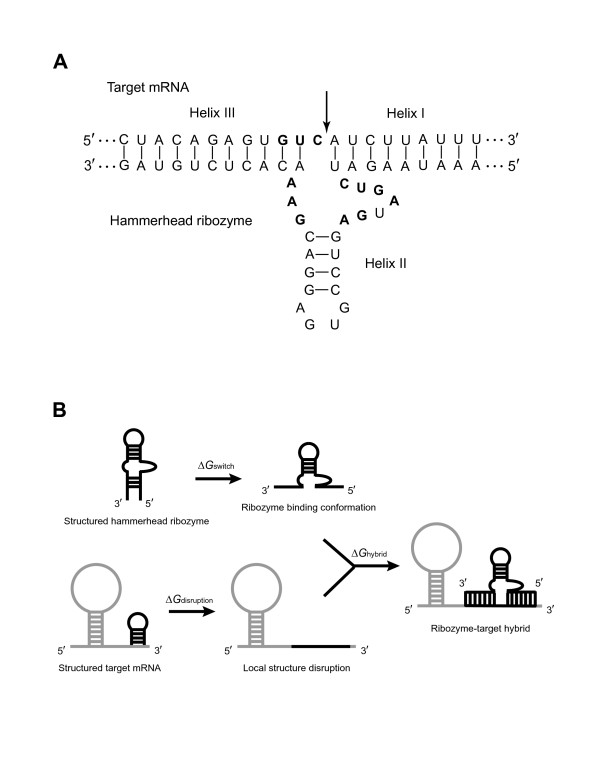
(**A**) Hammerhead ribozyme conformation arising from hybridization between hammerhead ribozyme GUC19 and the target (BCRP) mRNA. The GUC triplet in the target site and the conserved nucleotides in the ribozyme catalytic core are in boldface. The site of cleavage, i.e., 3' of the C of the GUC triplet, is indicated by an arrow. (**B**) Thermodynamic cycle of free energy exchanges. Δ*G*_disruption _is the target disruption energy, which represents the free energy cost to alter the local secondary structure at the target site for ribozyme binding; Δ*G*_switch _is the change in free energy from a predicted ribozyme conformation to the "active" binding conformation in which the catalytic core is correctly folded and both binding arms are single-stranded; Δ*G*_hybrid _is the free energy gain from the ribozyme-target hybridization (see Methods for description of calculations).

Ribozymes are short catalytic RNAs that possess the dual functions of sequence-specific RNA recognition and site-specific cleavage. For the self-cleaving (*cis*-acting) hammerhead ribozyme discovered by Haseloff and Gerlach [1], the binding arms at the 5' and 3' ends of the ribozyme form two helices, termed helix I and helix III, with the substrate. The catalytic core of the ribozyme contains helix II and largely conserved nucleotides. These structure and sequence features are illustrated in Figure 1A for the hammerhead conformation of a specific mRNA-targeting ribozyme studied here. For inhibition of the expression of a gene through targeting of the gene's mRNA, *trans*-cleaving hammerhead ribozymes can be engineered with binding arms whose sequences are complementary to the target mRNA sequences flanking a cleavage triplet NUH, where N is any nucleotide and H is any nucleotide except G. Among all possible NUH combinations, cleavage at GUC (see Figure 1A) has been reported to be the most effective [2].

In recent years, gene silencing by RNA interference (RNAi) [3] has emerged as a powerful tool for gene knockdown studies. However, applications of RNAi are limited to eukaryotes, whereas ribozymes can be applied in both eukaryotic and prokaryotic systems [4,5]. Furthermore, off-target effects by RNAi have been well noted [6-10]. Ribozymes, in contrast, generally possess high target specificity, owing to the dual requirements for cleavage, i.e., complementarity for the binding arms and a cleavage triplet. On the other hand, the knockdown effects by RNAi are stronger than those for ribozymes, suggesting that the two technologies can play complementary roles in functional genomics [11]. Ribozyme libraries constructed with randomized binding arms have been employed for successful identification of novel functional genes in mammalian cells [12-15].

The activities of *trans*-cleaving ribozymes can vary greatly for different sites on the same target mRNA. Such variability is considered to be largely due to differences in the accessibility of the target sites [11,16]. There is compelling evidence that, to a large extent, the secondary structure of an mRNA molecule determines the accessibility of the mRNA for numerous gene regulatory mechanisms that require complementary base-pairing for target recognition, including translational inhibition by antisense oligonucleotides [17], target cleavage by ribozymes [18] and siRNAs [19-23], and, more recently, repression of translation by microRNAs [24,25].

Experimental approaches to the identification of accessible target sites are tedious and time-consuming. The design of effective ribozymes presents a challenge and has largely been based on trial and error. Several computational methods [26-28] make accessibility predictions through structures predicted by the free energy minimization approach [29]. However, this method is not well suited to characterization of the likely population of structures that can exist in dynamic equilibrium *in vivo *for a specific mRNA molecule [30,31]. In recent years, an alternative sampling approach to RNA secondary structure prediction has been developed [32] and has been implemented as the Sfold program [33]. The structure sampling algorithm generates a statistically representative sample from the Boltzmann-weighted ensemble of RNA secondary structures for the RNA. In comparison to the minimum free energy method, this approach has been shown to better represent the likely population of mRNA structures [34], and to make improved predictions for structural RNAs [35]. Not surprisingly, predictions by Sfold significantly correlate with experimental results in gene down-regulation studies using antisense oligonucleotides [36,37], RNAi [38], or microRNAs [25]; in contrast, a lack of significant correlation was found in these applications for predictions based on free energy minimization. Here, we explore the potential value of using Sfold to predicting activities of hammerhead ribozymes.

In this study, a set of hammerhead ribozymes targeted to the transcript of the human ABCG2 gene encoding the breast cancer resistance protein (BCRP) were designed and analyzed *in vitro *(Table 1). We considered a number of structural and thermodynamic parameters that can affect the activity of a hammerhead ribozyme. Several of these parameters were computed with structures predicted by Sfold for the target mRNA and for the hammerhead ribozyme. One of the parameters measures the target accessibility, and is termed the target disruption energy; it represents the energy cost expended in altering the local target structure so as to allow ribozyme binding (Figure 1). We found that the target disruption energy and the total energy change of the ribozyme-target hybridization are two significant parameters for prediction of ribozyme activity. The target disruption energy is the major contributor to the predictability by the total energy change. In addition, for the ribozyme itself, incorrect folding of the catalytic core or interactions involving the two binding arms and the end sequences of the catalytic core can have detrimental effects on ribozyme activity. These findings suggest rules for a structure-based rational design of *trans*-cleaving hammerhead ribozymes.

**Table 1:** Hammerhead ribozymes targeted to 15 GUC cleavage sites in the BCRP mRNA

**Ribozyme**^*a*^	**Position of target site ^*b*^**	**Activity [(1-Su3600) ± SD]**	**Δ*G*_disruption_(kcal/mol)**	**Δ*G*_total _(kcal/mol)**
GUC1	-174	0.843 ± 0.047	-15.958	-5.423
GUC2	-131	0.890 ± 0.045	-11.898	-13.288
GUC3	+3	0.811 ± 0.116	-2.466	-17.571
GUC4	+16	0.803 ± 0.062	-11.090	-4.677
GUC5	+36	0.876 ± 0.030	-10.879	-9.883
GUC6	+164	0.802 ± 0.053	-17.339	-2.715
GUC7	+221	0.934 ± 0.025	-11.197	-16.294
GUC8	+274	0.844 ± 0.027	-7.312	-13.835
GUC9	+490	0.803 ± 0.030	-17.625	-6.215
GUC10	+506	0.829 ± 0.028	-15.457	-8.610
GUC11	+558	0.664 ± 0.067	-10.178	-26.245
GUC14	+1109	0.788 ± 0.066	-13.385	-5.469
GUC17	+1201	0.914 ± 0.067	-8.426	-11.931
GUC18	+1207	0.920 ± 0.042	-5.473	-17.366
GUC19	+1398	0.940 ± 0.047	-7.868	-9.736

^*a *^Ribozymes GUC12, GUC13, GUC15 and GUC16 were not tested.

^*b *^Position of G of the GUC cleavage site in the ABCG2 cDNA relative to the A of the ATG start codon (GenBank accession no. NM_004827).

## Results

### Measurement of ribozyme activity

**Figure 2:**
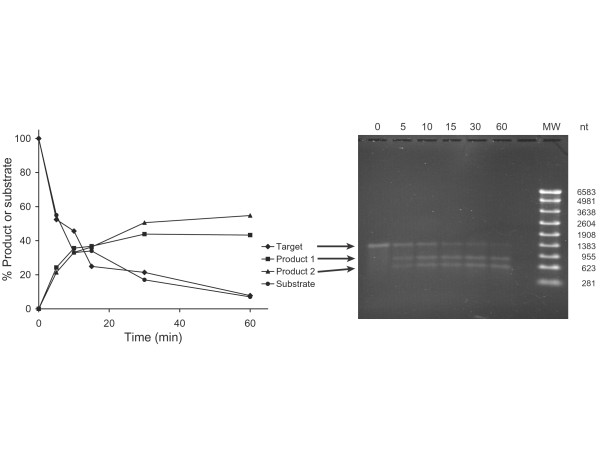
Comparison of methods for quantification of ribozyme cleavage. Ribozyme GUC7 was incubated for various lengths of time from 0 to 60 min, as indicated, and substrate cleavage activity was analyzed by agarose gel electrophoresis and real-time RT-PCR as described in Methods. After electrophoresis (right panel), the gel was stained with ethidium bromide, and the bands were quantified by densitometry. Relative band intensity was then graphed against time (left panel). Target (◆), remaining substrate; product 1 (■) and 2 (▲), relative amounts of each of the two cleavage products. Separately, the substrate was quantified by real-time RT-PCR, and the relative amount of remaining substrate (●) was graphed against time.

Traditionally, ribozyme activity is determined through *in vitro *cleavage followed by gel electrophoresis; the latter most often uses a radiolabeled substrate RNA combined with autoradiography [39], although non-radioactive detection by ethidium bromide staining has also been employed [40]. For both methods, quantification then requires densitometry of the cleavage products on the gel. Here, we wished to evaluate whether ribozyme cleavage activity can also be measured via quantitative RT-PCR. Accordingly, ribozyme GUC7 was incubated for varying lengths of time with the appropriate substrate RNA, and the remaining substrate was analyzed either by agarose gel electrophoresis followed by densitometry, or else by real time RT-PCR on the LightCycler. As shown by Figure 2, the results from these two methods are in good agreement. We concluded that quantitative RT-PCR is a valid method by which to determine ribozyme activity *in vitro*; thus, all activity measurements for our study were therefore made by this method.

### Statistical analyses

For the 15 ribozymes tested *in vitro*, we performed both correlation and regression analyses, using the ribozyme catalytic activity measured by (1-Su3600), and each of the computational parameters (see Methods). First, we observed that there were two outliers, namely, ribozymes GUC3 and GUC11, which behaved differently from the other 13 ribozymes. We thus initially focused on the analyses for the 13 "normally behaving" ribozymes, and then investigated possible explanations for the two outliers.

**Figure 3:**
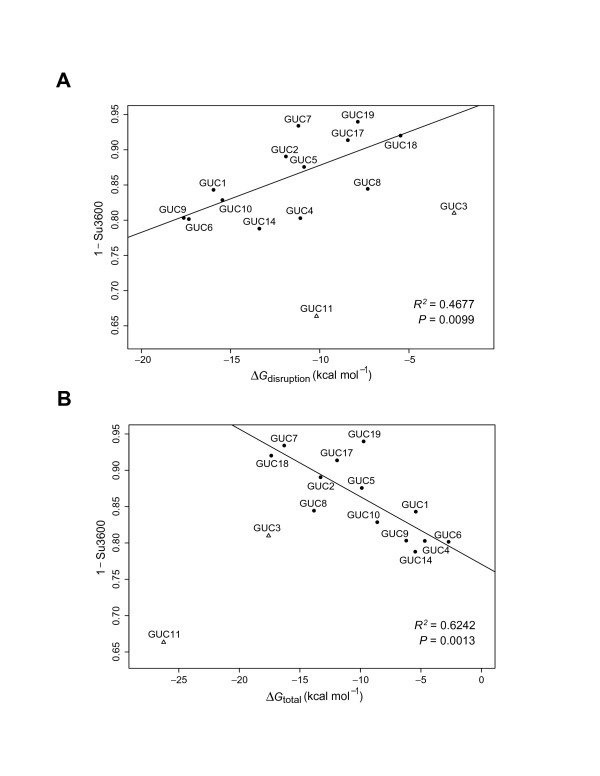
Linear regression for prediction of the ribozyme activity (as measured by (1-Su3600) for the amount of substrate cleaved at 1 hr) for 13 "normally behaving" ribozymes. (**A**) For Δ*G*_disruption _as the predictor, the *R*^2 ^for the regression is 0.4677, and the *P*-value is 0.0099. (**B**) For Δ*G*_total _as the predictor, the *R*^2 ^for the regression is 0.6242, and the *P*-value is 0.0013. Also plotted are the two outliers (GUC11 and GUC3) that were not included in the regression analysis (see Results, Figures 4 and 5 for explanations of the outliers).

For the 13 well behaved ribozymes, we found, among the structural and thermodynamic parameters, that Δ*G*_disruption _and Δ*G*_total _are significantly correlated with ribozyme activity (Table 2). The correlation coefficient for Δ*G*_disruption _is 0.6839 with a *P*-value of 0.0099, and the correlation coefficient for Δ*G*_total _is -0.7901 with a *P*-value of 0.0013. Ribozyme activity, however, was not significantly correlated with Δ*G*_switch_, or Δ*G*_hybrid_. Because Δ*G*_total _is computed from Δ*G*_disruption_, Δ*G*_switch_, and Δ*G*_hybrid _(see Methods), Δ*G*_total _and Δ*G*_disruption _are significantly correlated (correlation coefficient = -0.6349, and *P*-value = 0.0110). Thus, the significance of the correlation with the ribozyme activity for Δ*G*_total _is mainly due to Δ*G*_disruption_. From linear regression analysis, either Δ*G*_disruption _or Δ*G*_total _is significantly predictive of the ribozyme activity (Table 2, Figure 3). Furthermore, in a comparison of the *R*^2 ^values for Δ*G*_disruption _and Δ*G*_total_, a relative improvement of about 33.5% is observed for Δ*G*_total_. This suggests that, although Δ*G*_switch _and Δ*G*_hybrid _are insignificant as individual predictors, they do contribute to the improved predictability by Δ*G*_total_. The *R*^2 ^for Δ*G*_total _indicates that over 60% of the variability in the ribozyme cleavage activity can be attributed to Δ*G*_total_.

**Table 2:** Linear regression and correlation analyses ^*a*^

**Parameter**	**Linear Regression**			**Correlation coefficient**
	
	**Coefficient**	***P*-value**	**R**^2^	
**Δ*G***_disruption_	0.0095	0.0099	0.4677	0.6839
**Δ*G***_switch_	0.0024	0.8616	0.0029	0.0537
**Δ*G***_hybrid_	-0.0048	0.3498	0.0798	-0.2825
**Δ*G***_total_	-0.0093	0.0013	0.6242	-0.7901

^*a *^Two outliers, GUC3 and GUC11 (see Figure 3) were excluded in these analyses

**Figure 4:**
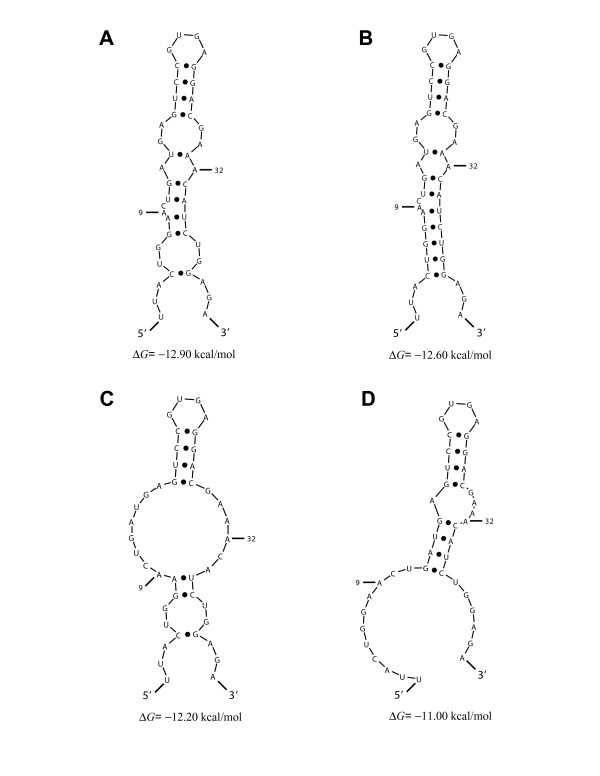
Structural analysis of GUC3, one of the two outliers in Figure 3. Unfavorable interactions involving the two binding arms and the end sequences of the ribozyme catalytic core are present in structures predicted for GUC3. (**A**) The representative structure (i.e., the centroid of a structural cluster [35]) for 44.9% of structures predicted by Sfold for the ribozyme sequence. (**B**) The representative structure for 29.8% of the predicted structures. (**C**) The representative structure for 20.9% of the predicted structures. (**D**) The representative structure for the remaining 4.4% of the predicted structures. The sequence for the ribozyme 5' binding arm ends at A^9^, and the sequence for the ribozyme 3' binding arm starts at A^32^.

**Figure 5:**
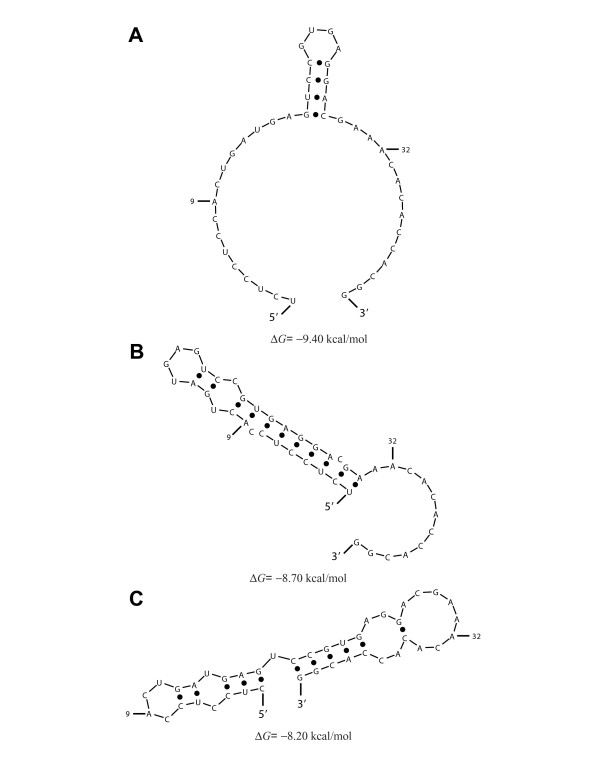
Structural analysis of GUC11, the other outlier in Figure 3. For a substantial portion of the structure sample generated by Sfold for GUC11, the predicted structure has a misfolded catalytic core. (**A**) Ribozyme in "active" binding conformation with correctly folded core (i.e., helix II and single stranded end sequences of the core including 9 conserved nucleotides, as shown in Figure 1A), representing 66.5% of the predicted structures. (**B**) A conformation with a misfolded core, representing 28% of the predicted structures. (**C**) Another conformation with a misfolded core, representing 5.5% of the predicted structures. The sequence for the ribozyme 5' binding arm ends at A^9^, and the sequence for the ribozyme 3' binding arm starts at A^32^.

To understand the behaviors of the two outliers, ribozymes GUC3 and GUC11, we examined the structures predicted by Sfold for each of the ribozymes. In the case of ribozyme GUC3, we found that, for 79.1% of the structures, there are at least four base pairs formed by nucleotides in the two binding arms and the ends of the catalytic core sequence (Figure 4). In the "active" ribozyme binding conformation (Figure 1B), all of not only the binding arms but also the end sequences of the catalytic core are single-stranded. Thus, substantial intramolecular structure involving these regions can hinder target binding by the ribozyme, despite the correct formation of helix II (Fig 1A). For GUC11, we found that 33.5% of sampled structures have the catalytic core misfolded so that it lacks a correctly formed helix II (Figure 5). This could explain the observation that GUC11 was the least effective for target cleavage (Table 1, Figure 3), despite a moderately accessible target site as indicated by Δ*G*_disruption _(Table 1). In contrast, for each of the other 14 ribozymes in our study, the percentage of the sampled structures having a misfolded core is less than 1%.

## Discussion and conclusion

In this work, we have considered a number of structural and thermodynamic parameters and have assessed their effects on the *in vitro *activities of *trans*-cleaving hammerhead ribozymes. We found that Δ*G*_disruption_, a measure of accessibility at the target site, and Δ*G*_total_, a measure of the total energy change for the ribozyme-target hybridization process, are two significant parameters for predicting ribozyme activity, and that Δ*G*_disruption _is the major contributor to the predictability by Δ*G*_total_. In this analysis, the stability of the ribozyme-target hybrid as measured by Δ*G*_hybrid _had no impact on ribozyme cleavage activity. Furthermore, we found that incorrect folding of the ribozyme catalytic core or interactions involving the two binding arms and the end sequences of the catalytic core can have detrimental effects on ribozyme activity.

For the selection of functional ribozymes for gene knock-down studies, it is advisable to choose highly accessible target sites (i.e., sites with favorable Δ*G*_disruption_) and favorable Δ*G*_total_. In addition, a ribozyme with either a misfolded core or structures that are substantially different from the "active" binding conformation (Figure 1B) should be avoided.

Our analyses are limited to modeling of RNA secondary structures. The *R*^2 ^of 62.42% for Δ*G*_total _indicates that the remaining 37.58% of the variability in the ribozyme cleavage activity must be attributed to other factors that likely include RNA tertiary interactions [41].

We adopted a population approach to modeling of mRNA secondary structure, by employing the Sfold program. In an antisense application, predictions by Sfold were significantly correlated with activity of antisense oligonucleotides, whereas there was a lack of correlation for the minimum free energy (MFE) predictions [36]. In an RNAi application, the Sfold predictions were significantly predictive of RNAi efficacy [38]. In contrast, the predictive significance was either lacking or poor in terms of *R*^2 ^for predictions by MFE structures, by heuristic suboptimal foldings [42], or by complete suboptimal foldings [43]. Furthermore, for all of these RNA folding programs, only Sfold predictions were found to fully explain sensitivity of *lin-41 *mutants to microRNA repression by *let-7 *[25]. In the current application, if we employ the minimum free energy predictions by mfold [29] in the calculation of Δ*G*_disruption _and Δ*G*_switch_, we observe an insignificant correlation with the ribozyme activity for Δ*G*_disruption _(*P*-value = 0.0730, *R*^2 ^= 0.2632, and correlation coefficient = 0.5130), and substantially reduced significance and predictability for Δ*G*_total _(*P*-value = 0.0277, *R*^2 ^= 0.3687, and correlation coefficient = -0.6072). This finding further validates the sampling approach to characterization of the likely population of mRNA structures.

In the calculation of Δ*G*_disruption_, we assumed that the binding of target mRNA by a ribozyme induces only a local structural alteration at the target site. It is likely that in some, if not all, cases, nucleotides outside the target site will also contribute to the energy change due to ribozyme binding. An alternative to the local disruption model is a global disruption model, which assumes that the rest of the target mRNA molecule can refold after ribozyme binding. For this model, Δ*G*_disruption _can be re-calculated by constraining the target site to be single-stranded and refolding the rest of the target mRNA. Surprisingly, we observed insignificant correlation and poor predictability either for Δ*G*_disruption _(*P*-value = 0.7206, *R*^2 ^= 0.0121, and correlation coefficient = 0.1100), or for Δ*G*_total _(*P*-value = 0.3039, *R*^2 ^= 0.0956, and correlation coefficient = -0.3093). These results suggest that target cleavage occurs rapidly after the completion of ribozyme-target hybridization such that global refolding of the target before cleavage is unlikely. While partial refolding is a possibility, it is highly uncertain what region of the target will be involved in refolding. Thus, it is difficult to construct a computational model that can represent a reasonable compromise between the local model and the global model.

When the standard deviations for the measured activities are available, an alternative to the standard linear regression analysis is the weighted regression analysis. In a weighted least-squares regression, the square term in the sum of squares for a data point is multiplied by a weight [44]. In general, a larger weight is assigned to a data point with a higher precision as indicated by a smaller standard deviation. Specifically, with the standard deviation of cleavage activity available for every ribozyme (Table 1), 1/(standard deviation)^2^, that is, 1/variance, can be used as the weight [44]. The weighted regression yielded results that are highly similar to those from the un-weighted regression analysis. For example, for Δ*G*_total_, the *R*^2 ^is 0.6119, with a *P*-value of 0.0016.

While we focused on GUC cleavage sites in the present study, we have also tested a small set of non-GUC NUH sites. We observed a similar level of correlation between ribozyme activity and Δ*G*_disruption _(data not shown), suggesting that the critical parameters identified here are generally relevant for the prediction of the activity of hammerhead ribozymes. However, the levels of activities for non-GUC sites were generally lower than those for the GUC sites, consistent with a previous report that GUC is the most effective target site [2]. To generalize our findings for GUC target sites, further *in vitro *testing using other targets, as well as *in vivo *testing in cultured cells, will be required. The latter is currently in progress for the ribozymes described here.

## Methods

### Preparation of double-stranded DNA oligonucleotide ribozyme templates

For creation of the ribozymes *in vitro*, two complementary oligonucleotides containing the hammerhead ribozyme core sequence flanked by the sequences for the two binding arms and a T7 RNA polymerase promoter sequence were annealed into duplex DNA in 10 mM Tris, pH 8.0, and 50 mM NaCl, by incubation at 94°C for 5 min, followed by slow cooling to room temperature. All oligonucleotides were obtained from Integrated DNA Technology (IDT, Coralville, IA).

### *In vitro *transcription of ribozyme and substrate target RNA

*In vitro *transcription of the substrate and ribozyme RNA was performed using the MEGAscript and MEGAshortscript kits (Ambion-ABI, Austin, TX), respectively, following the manufacturer's instructions. Either 2.5 μg of linearized plasmid (pTRIamp19, Ambion) containing the target ABCG2 cDNA sequence, or 1.5 μg of ribozyme DNA were used as template. After transcription, the DNA templates were digested with RQ1 RNase-free DNase. Unincorporated nucleotides were removed from the RNA transcripts by size-exclusion chromatography with a ProbeQuant G-50 Micro Column (GE-Healthcare, Piscataway, NJ) or by phenol/chloroform extraction, both of which were followed by an ethanol precipitation. The purified *in vitro*-transcribed RNAs and ribozymes were then quantitated spectrophotometrically, and their quality verified by gel electrophoresis (see Additional file 1). Two separate substrate RNAs were made, one from nucleotides -225 to +1011, and one from nucleotides +586 to +1708, relative to the A of the ATG start codon of the full length ABCG2 cDNA (GenBank accession no. NM_004827). Individual ribozymes are numbered consecutively in the order of occurrence of the GUC cleavage sites to which they bind, starting from nucleotide -285. Thus, for example, GUC1 refers to the first GUC triplet after nucleotide -285. A total of 15 hammerhead ribozymes targeted to GUC sites were designed and prepared (Table 1). These ribozymes were constructed with the same ribozyme core sequence, but with different sequences for binding arms that were complementary to the target sequences at the binding site (Figure 1A; also see Additional file 2). For each of these ribozymes, the 3' binding arm had 11 nucleotides, and the 5' binding arm had nine nucleotides.

### *In vitro *cleavage of target sequence and identification of cleavage products

The target RNA (10 pmol) and ribozyme (50 pmol) under study were mixed in 50 mM Tris, pH 8.0, and the *in vitro *cleavage reaction was initiated by the addition of 20 mM MgCl_2_. One μl of RNaseGuard was also added, and the mixture was incubated at 37°C. 10-μl aliquots were removed after 0, 5, 10, 15, 30, and 60 min, and the reaction was terminated by the addition of 50 mM EDTA. The cleavage products were then analyzed by electrophoresis in a 2% (v/v) formaldehyde/2.0% (w/v) agarose gel for 3–4 hr at 70 V. The separated products were stained with SYBR Green or ethidium bromide and photographed under UV light [40].

### Quantification of residual substrate by real-time RT-PCR

Since a ribozyme irreversibly cleaves its substrate, we reasoned that the cleavage reaction could be quantified through measurement of the amount of substrate remaining by real-time RT-PCR, using primer pairs that span the cleavage site. Accordingly, an aliquot of the cleavage reaction containing both the remaining, uncleaved substrate and the cleavage products was added to a one-step real-time RT-PCR reaction mix containing SYBR Green (Sigma, St. Louis, MO) according to the manufacturer's instructions, and amplification was carried out for 35–45 cycles in a LightCycler^® ^(Roche, Indianapolis, IN), under conditions appropriate for each primer pair (see Additional file 3). Primers flanking each cleavage site were chosen such that the PCR products were between 600 and 400 bp long. The amount of uncleaved substrate present was determined from the crossing point values (C_T_) calculated by the Lightcycler software from the amplification curve. The relative amount of template remaining at each time point (Su(t)) was then calculated by 2−(CT(t)−CT(0))
 MathType@MTEF@5@5@+=feaafiart1ev1aaatCvAUfKttLearuWrP9MDH5MBPbIqV92AaeXatLxBI9gBaebbnrfifHhDYfgasaacPC6xNi=xH8viVGI8Gi=hEeeu0xXdbba9frFj0xb9qqpG0dXdb9aspeI8k8fiI+fsY=rqGqVepae9pg0db9vqaiVgFr0xfr=xfr=xc9adbaqaaeGacaGaaiaabeqaaeqabiWaaaGcbaGaeGOmaiZaaWbaaSqabeaacqGHsislcqGGOaakcqqGdbWqdaWgaaadbaGaeeivaqLaeiikaGIaeeiDaqNaeiykaKcabeaaliabgkHiTiabboeadnaaBaaameaacqqGubavcqGGOaakcqaIWaamcqGGPaqkaeqaaSGaeiykaKcaaaaa@3B27@, where C_T(t) _is the C_T _value at time t, and C_T(0) _is the C_T _value at time 0. Each time point was assayed in duplicate, and each cleavage reaction was repeated at least four times independently with different batches of substrate RNA. Selected ribozymes were also analyzed with differing ribozyme preparations. No significant activity differences were observed between separate ribozyme and/or substrate preparations. For the subsequent calculations, the relative amount of substrate cleaved at 3600 sec (1-Su3600) was used as the measure of ribozyme activity. In preliminary experiments, we determined that the RT-PCR reaction was linear with the amount of substrate present (data not shown).

### Prediction of mRNA secondary structure

The determination of mRNA secondary structure presents both theoretical and experimental challenges. One major impediment to the accurate prediction of mRNA structures stems from the likelihood that a specific mRNA molecule does not adopt a single structure in solution, but instead likely exists in thermodynamic equilibrium among a population of structures [30,31,45]. Thus, the computational prediction of secondary structure based on free energy minimization is not well suited to the task of providing a realistic representation of mRNA structures *in vivo*.

An alternative to free energy minimization for characterization of the ensemble of probable structures for a given RNA molecule has been developed [32]. In this approach, a statistically representative sample is drawn from the Boltzmann-weighted ensemble of RNA secondary structures for the RNA. Such samples of even moderate size can faithfully and reproducibly characterize structure ensembles of enormous size, so that sampling estimates of structural features are statistically reproducible from one sample to another. In particular, in comparison to free-energy minimization, this method has been shown to make better structural predictions [35] and to better represent the likely population of mRNA structures [34], and to yield a significant correlation between predictions and antisense inhibition data [36,37]. A sample size of 1,000 structures has been shown to be sufficient to guarantee statistical reproducibility in typical sampling statistics and structure clustering features [32,34]. In applications to modeling RNA target binding by a (partially) complementary nucleic acids, because a single-stranded block of four or five nucleotides is essential for the nucleation step of the hybridization [25,46,47], the probability that such block is single-stranded must be high. Thus, in the current and other related applications, we consider the sample size of 1,000 to be sufficient. In the case that a structural feature of small probability is of interest, a much larger sample would be required. The structure sampling method has been implemented in the Sfold software program for RNA folding and applications [33] and is used here for mRNA folding.

### Prediction of ribozyme secondary structure

The core of the ribozyme is considered to exist in a mixture of conformations in solution that can interchange rapidly [48-51]. In accordance with this established dynamic view of the hammerhead structure, we also employed Boltzmann structure samples generated by Sfold for the prediction of ribozyme secondary structure. Again, a sample size of 1,000 was used for characterizing probable ribozyme structures at equilibrium.

### Structural and thermodynamic parameters

The catalytic activity of a *trans*-cleaving ribozyme can be affected by many factors. Here, we have focused on a number of structural and thermodynamic parameters. These parameters take into account the secondary structure of the target, the secondary structure of the ribozyme, and the stability of the ribozyme-target duplex. Below, we define these terms in the current context and compute the total free energy change for modeling the hybridization process.

Δ*G*_disruption _is the free energy cost for disruption of the secondary structure at the ribozyme binding site on the target mRNA (Figure 1B), and thus is a measure of accessibility at the target site. For the 15 designed ribozymes, each with nine nucleotides for the 5' binding arm and 11 nucleotides for the 3' binding arm, the binding site involves 20 nucleotides, excluding the unpaired C of the GUC triplet (Figure 1A). To calculate Δ*G*_disruption_, we adopted the simplifying assumption that the binding of a ribozyme to a relatively much longer mRNA should induce a local structural alteration at the target site, but no longer-range effects on overall target secondary structure. In other words, we defined local structural alteration as the breakage of the intramolecular base pairs involving the target site to permit formation of the ribozyme-target duplex (Figure 1). Specifically, Δ*G*_disruption _was calculated as the energy difference between Δ*G*_before_, the free energy of the original mRNA structure, and Δ*G*_after_, the free energy of the new, locally altered structure (Δ*G*_disruption _= Δ*G*_before_- Δ*G*_after_). We calculated Δ*G*_before _from the average energy of the original 1,000 structures predicted by Sfold, and Δ*G*_after _from the average energy of all of the 1,000 locally altered structures. Therefore, under the local disruption assumption, the calculations did not require refolding of the rest of the target sequence.

Δ*G*_switch _is the free energy cost for the ribozyme to switch from one conformation to the conformation that is most favorable for target binding and subsequent cleavage. Here, the starting conformation is any conformation predicted by Sfold, and the binding conformation is the one for which the ribozyme core is correctly folded and both binding arms are single-stranded (Figure 1B). Thus, Δ*G*_switch _= Δ*G*_s _- Δ*G*_b_ , where Δ*G*_s_ is the free energy of the starting conformation, and Δ*G*_b_ is the free energy of the binding conformation. In the case that the starting conformation is the binding conformation, Δ*G*_switch _= 0.0 kcal/mol. We calculated Δ*G*_s _by the average free energy of the 1000 structures predicted by Sfold for the ribozyme. Δ*G*_b _is the same for different starting conformations of a given ribozyme sequence, so there is no need to average over a structure sample.

Δ*G*_hybrid _is the energy gain due to the complete intermolecular hybridization between the ribozyme binding arms and the nucleotide sequence of the target binding site. It is calculated by the sum of base-pair stack energies for the two ribozyme arm-target duplexes, an energetic penalty ("initiation energy") for the initialization of bimolecular interaction [52], and other penalties or energies associated with the multi-branched loop formed by the three adjacent helices. Specifically, Δ*G*_hybrid _= Δ*G*_initiation _+ ∑_1≤*i*≤10_Δ*G*_H3_stacking(i) _+ ∑_1__≤*j*≤8_Δ*G*_H1_stacking (*j*) _+ Δ*G*_multi-loop_+ Δ*G*_H3_terminal _+ Δ*G*_H1_terminal _+ Δ*G*_dangle_, where the initiation energy Δ*G*_initiation _= 4.1 kcal/mol [52]; Δ*G*_H3_stacking (*i*) _(1 ≤ *i *≤ 10) is the stacking energy for the *i*-th base-pair stack for helix III (Figure 1A); Δ*G*_H1_stacking (*j*) _(1 ≤ *j *≤ 8) is the stacking energy for the *j*-th base-pair stack for helix I; Δ*G*_multi-loop _is a linear penalty for the multibranched loop formed by the three helices; Δ*G*_H3_terminal _is a penalty of 0.5 kcal/mole for the terminal A-U pair for helix III, while Δ*G*_H1_terminal _applies the same penalty for a terminal A-U or G-U pair [53] for helix I (e.g., A-U for ribozyme GUC19, Figure 1A); and Δ*G*_dangle _is a sum of free energies for dangling ends (i.e., single base stacks)[52]. More specifically, for the linear multibranched loop penalty, Δ*G*_multi-loop_= *a *+ *b*(number of unpaired bases)+*c*(number of helices), where *a*, *b*, and *c *are respectively the offset, the free base penalty and the helix penalty, and *a *= 3.4 kcal/mol, *b *= 0.0 kcal/mol, and *c *= 0.4 kcal/mol [53]. In our present context, there are 11 unpaired bases and three helices in the loop, so Δ*G*_multi-loop_= 5.2 kcal/mol, a constant for all ribozymes studied here. For a terminal base-pair N-N' (A-U for ribozyme GUC19, as shown in Figure 1A) for helix I, Δ*G*_dangle _= min [Δ*G*_3(*U*-*A*,*C*)_, Δ*G*_5(*N*-*N*',*C*)_] + Δ*G*_5(*A*-*U*,*A*) _+ Δ*G*_3(*C*-*G*,*G*) _+ Δ*G*_5(*G*-*C*,*A*) _+ Δ*G*_3(*N*'-*N*,*C*)_, where the free energies for both 5' and 3' dangling ends [53] are used, and min [Δ*G*_3(*U*-*A*,*C*)_, Δ*G*_5(*N*-*N*',*C*) _] is the minimum of the two dangling energies, to take into account two possibilities of single-base stacking for the C of the GUC cleavage triplet. It is assumed that a single unpaired nucleotide between two adjacent helices for a multi-branched loop stacks onto the terminal base pair of the helix possessing the more favorable dangling energy.

Finally, we computed Δ*G*_total_, the total energy change for the ribozyme-target hybridization. Δ*G*_total _can be calculated through consideration of the energy gain due to the complete intermolecular hybridization and the energy costs owing to structure alterations for both the target and the ribozyme. With use of the parameters introduced above, Δ*G*_total _= Δ*G*_hybrid _- Δ*G*_switch _- Δ*G*_disruption_.

### Statistical analyses

The standard univariate linear regression was used for predicting ribozyme activity by each of the parameters listed above. The *P*-value measures the statistical significance of the parameter, and the *R*^2 ^of the regression indicates the degree of variability in ribozyme activity that is attributed to the parameter. The Pearson's correlation coefficient between a parameter and the ribozyme activity was also computed. We note that the *P*-value of the correlation is the same as the *P*-value of the parameter from the standard univariate regression analysis. The software package R [54] was used for the statistical analyses.

## Availability and requirements

The energetic calculations in this study have been incorporated into the Sribo module of the Sfold program. Sribo is freely available for academic applications through a Web server at . Structural predictions and features are also available from the Srna module of Sfold . For commercial usage, a license is required .

## Competing interests

The author(s) declare that they have no competing interests.

## Authors' contributions

YD and ES conceived the study. YS and CYC performed computational analyses. SW and JRK carried out the *in vitro *experiments. YD supervised the computational work. ES supervised the experimental work. YS, YD and ES wrote the manuscript. All authors read and approved the final manuscript.

## Supplementary Material

Additional File 1 A representative gel. *In-vitro *transcribed and purified ribozymes were analyzed by denaturing gel electrophoresis. The figure shows a representative gel with four different ribozymes. Click here for file

Additional File 2 Sequences of hammerhead ribozymes. The table lists sequences of 15 hammerhead ribozymes targeted to the BCRP mRNA. Click here for file

Additional File 3 Primer sequences for RT-PCR amplification. The table lists primer sequences for RT-PCR amplification of the ribozymes for quantifying cleavage reaction. Click here for file
