# Supplementary material for: A structural analysis of in vitro catalytic activities of hammerhead ribozymes
Source: BMC Bioinformatics. 2007 Nov 30;8:469. doi: 10.1186/1471-2105-8-469 (PMC2238771; doi:10.1186/1471-2105-8-469)
Supplement: Additional File 1 — A representative gel. In-vitro transcribed and purified ribozymes were analyzed by denaturing gel electrophoresis. The figure shows a representative gel with four different ribozymes. [file 1471-2105-8-469-S1.pdf]

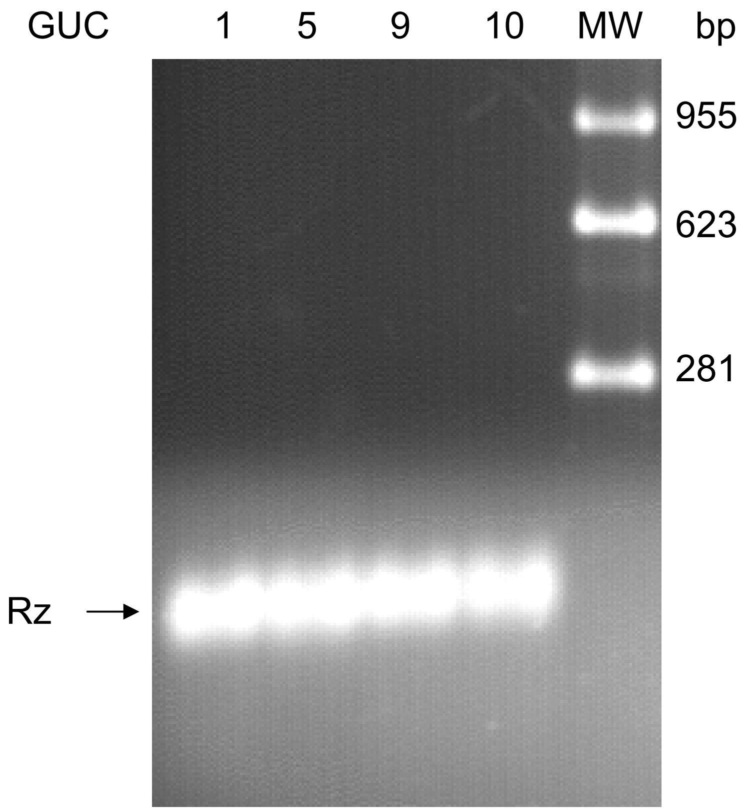

Supplementary Figure 1: *In-vitro* transcribed and purified ribozymes were analyzed by denaturing gel electrophoresis. A representative gel with four different ribozymes is shown.
