# Supplementary material for: A structural analysis of in vitro catalytic activities of hammerhead ribozymes
Source: BMC Bioinformatics. 2007 Nov 30;8:469. doi: 10.1186/1471-2105-8-469 (PMC2238771; doi:10.1186/1471-2105-8-469)
Supplement: Additional File 2 — Sequences of hammerhead ribozymes. The table lists sequences of 15 hammerhead ribozymes targeted to the BCRP mRNA. [file 1471-2105-8-469-S2.doc]

| **Supplementary Table 1**. Sequences of 15 hammerhead ribozymes targeted to BCRP mRNA | |
| --- | --- |
| Name | Sequence (5′ → 3′) |
| **GUC1** | **ACU UAA CAA CUG AUG AGU CCG UGA GGA CGA A Ac CAC CAA GCA** |
| **GUC2** | **AGU CAC CCG CUG AUG AGU CCG UGA GGA CGA A Ac CUU CCA AAC** |
| **GUC3** | **UUA CUG GAA CUG AUG AGU CCG UGA GGA CGA A Ac AUC UGG AGA** |
| **GUC4** | **AAA AAC UUC CUG AUG AGU CCG UGA GGA CGA A Ac AUU ACU GGA** |
| **GUC5** | **UUU CCU UGU CUG AUG AGU CCG UGA GGA CGA A Ac ACU GGG AUA** |
| **GUC6** | **CUG GUU UUC CUG AUG AGU CCG UGA GGA CGA A Ac AAG GUA GAA** |
| **GUC7** | **UGG CGU UGA CUG AUG AGU CCG UGA GGA CGA A Ac CAG GUU Uca** |
| **GUC8** | **UGC AGC UAA CUG AUG AGU CCG UGA GGA CGA A Ac AUC UAA UAA** |
| **GUC9** | **CUC UUG AAU CUG AUG AGU CCG UGA GGA CGA A Ac CCU GUU AAU** |
| **GUC10** | **CUU UAU CCA CUG AUG AGU CCG UGA GGA CGA A Ac CUA ACU CUU** |
| **GUC11** | **UCU CCU CCA CUG AUG AGU CCG UGA GGA CGA A Ac ACA CCA CGG** |
| **GUC14** | **GGA GGA GUU CUG AUG AGU CCG UGA GGA CGA A Ac AUA AAU CUC** |
| **GUC17** | **Uac gac UgU CUG AUG AGU CCG UGA GGA CGA A Ac aaU gaU cUg** |
| **GUC18** | **Ucc cag Uac CUG AUG AGU CCG UGA GGA CGA A Ac UgU gac aaU** |
| **GUC19** | **AAA UAA GAU CUG AUG AGU CCG UGA GGA CGA A Ac ACU CUG UAG** |
| The nucleotides in the ribozyme catalytic core, which are identical for all ribozymes, are in black. The nucleotides in the binding arms, which are target-site specific, are in blue. | |
