# Supplementary material for: A structural analysis of in vitro catalytic activities of hammerhead ribozymes
Source: BMC Bioinformatics. 2007 Nov 30;8:469. doi: 10.1186/1471-2105-8-469 (PMC2238771; doi:10.1186/1471-2105-8-469)
Supplement: Additional File 3 — Primer sequences for RT-PCR amplification. The table lists primer sequences for RT-PCR amplification of the ribozymes for quantifying cleavage reaction. [file 1471-2105-8-469-S3.pdf]

Supplementary Table 2. Ribozyme primer sequences

Rz	Upper primer sequence	Location*	Lower primer sequence	Location*	Substrate	Annealing	Extension	Acquisition	# Cycles
						Temp (°C)/Time (s)			
**GUC1**	5'-CTG TGG AGG AAC TGG GTA GG-3'	-225 to -206	5'-TAA CGA AGA TTT GCC TCC ACC TGT G-3'	266 to 242	**5'**	63/5	72/20	81/5	35
**GUC2**					**5'**				
**GUC3**	5'-GAC TCA TCC CAA CAT TTA CAT CC-3'	-123 to -101	5'-TAA CGA AGA TTT GCC TCC ACC TGT G-3'	266 to 242	**5'**	65/5	72/20	80/5	40
**GUC4**					**5'**				
**GUC5**					**5'**				
**GUC6**					**5'**				
**GUC7**					**5'**				
**GUC8**	5'-GGT GGA GGC AAA TCT TC-3'	246 to 262	5'-AGT CCT TTT TCT TTC TCC-3'	581 to 564	**5'**	55/5	72/20	80/5	40
**GUC9**					**5'**				
**GUC10**					**5'**				
**GUC11**					**5'**				
**GUC12**	5'-ATC CTT CCA TCT TGT TCT-3'	607 to 624	5'-AGA AGG AGG AGT TGA CAT-3'	1023 to 1006	**3'**	60/5	72/20	80/5	45
**GUC13**					**3'**				
**GUC14**	5'-CAA GCA GGA TAA GCC ACT CAT AG-3'	965 to 987	5'-GGC TGA AAC ACT GCT GAA ACA CTG-3'	1331 to 1308	**3'**	63/5	72/20	81/5	35
**GUC15**					**3'**				
**GUC16**	5'-GTC TTC AAG GAG ATC AGC TAC ACC-3'	1086 to 1109	5'-GCT GAA TAA GCC ACC ATC ATA AGG-3'	1558 to 1535	**3'**	63/5	72/20	80/5	40
**GUC17**					**3'**				
**GUC18**					**3'**				
**GUC19**					**3'**				

* Location of primers is based on #1 = the A of ATG
